# West Nile Virus Infection Incidence Based on Donated Blood Samples and Neuroinvasive Disease Reports, Northern Texas, USA, 2012

**DOI:** 10.3201/eid2104.141178

**Published:** 2015-04

**Authors:** Diana T. Cervantes, Shande Chen, Laurie J. Sutor, Shelley Stonecipher, Nicolette Janoski, David J. Wright, Michael P. Busch

**Affiliations:** Texas Department of State Health Services, Arlington, Texas, USA (D.T. Cervantes, S. Stonecipher);; The University of North Texas Health Science Center, Fort Worth, Texas, USA (S. Chen);; Carter BloodCare, Bedford, Texas, USA (L.J. Sutor);; Tarrant County Public Health, Fort Worth (N. Janoski);; Westat, Rockville, Maryland, USA (D. Wright);; Blood Systems Research Institute, San Francisco, California, USA (M.P. Busch)

**Keywords:** West Nile virus, WNV, viruses, West Nile neuroinvasive disease, zoonoses, vector-borne infections, transfusions, antibodies, blood donors, Texas

## Abstract

During the 2012 outbreak of West Nile virus in the United States, approximately one third of the cases were in Texas. Of those, about half occurred in northern Texas. Models based on infected blood donors and persons with neuroinvasive disease showed, respectively, that ≈0.72% and 1.98% of persons in northern Texas became infected.

From the first reported cases of West Nile Virus (WNV) in North America in August of 1999 through 2013, more than 39,000 cases of West Nile virus (WNV) were reported in the United States (*1*). In 2003, identification of transfusion-transmitted WNV infections (*2*) led to screening of the blood supply for WNV by using nucleic acid amplification technology (NAT) assays in mini-pools (MP-NAT) (*3*). Despite the success of MP-NAT screening of samples from blood donors, WNV transmission from infected donors continued. During 2004, screening algorithms expanded, including triggered individual donation NAT (ID-NAT) (*3*). Approximately 25% of viremic blood donors can be detected by ID-NAT (*4*).

Estimates of WNV infections in 2003 were derived from viremic blood donor rates detected by MP-NAT throughout the United States. West Nile neuroinvasive disease (WNND) reports were then used to approximate the number of infections relative to WNND cases (*5*). With the introduction of targeted ID-NAT, estimates of WNV infections from viremic blood donors must account for differential ID-NAT and MP-NAT screening during epidemic seasons.

Nationwide, the largest WNV epidemic since 2003 occurred in 2012, and approximately one third of cases were reported from Texas. Approximately 48% of cases in Texas were in 4 counties: Collin, Dallas, Denton, and Tarrant, located in the northern area of the state. The aim of this study was to estimate the number of WNV infections in this area during the 2012 arboviral season using 2 models: blood donor NAT yield and WNND-based models (*5*,*6*).

## The Study

Counts of screened blood donations and confirmed WNV viremic donations detected by MP-NAT or ID-NAT from northern Texas residents during the WNV season (April 1, 2012, through November 30, 2012, the WNV surveillance period used by AABB for triggering ID-NAT screening [*4*]) were obtained from Carter BloodCare and Creative Testing Solutions, the area blood collection organization and donor screening laboratory, respectively. Carter BloodCare accounts for >95% of blood donation centers in the North Texas Region. These data were used to derive the WNV seasonal incidence rate in 2012.

Calculations were performed separately for ID-NAT– and MP-NAT–screened donations. The length of time WNV RNA is detectable by MP-NAT has been previously reported (*7*). For this analysis, we derived new estimates for the duration of the MP-NAT and ID-NAT detection periods (Technical Appendix).

WNV seasonal incidence rates were obtained using a previously derived formula (*5*) by using rates of detection and durations of ID-NAT and MP-NAT WNV RNA detection periods. CIs were obtained assuming a Poisson distribution for ID-NAT and MP-NAT yields. The WNV seasonal incidence rate in blood donors and days screened per method were then applied to the estimated 2011 population of the 4 counties who were age-eligible for blood donation (>16 years of age) (*8*) to estimate the number of WNV infections in that area during the 2012 WNV season.

To estimate the number of WNV infections by age and gender, we used confirmed and probable WNND cases in persons >16 years of age reported during the WNV season to the Texas Department of State Health Services and included in ArboNET, a national surveillance system which monitors WNV activity (*6*). CIs were obtained by applying Taylor series expansion (*9*), based on a Poisson distribution for the WNND cases and the estimated variance of the ratio of WNV cases to WNND cases as reported (*6*).

## Results

Fifty-four WNV viremic donations were detected: 30 by MP-NAT and 24 by ID-NAT (Table 1). Dividing the number of viremic donations detected by donations screened by each method, 2.5 WNV-confirmed RNA-positive donations (MP-NAT screening periods) and 15.9 WNV-confirmed RNA-positive donations (ID-NAT screening periods) were detected per 10,000 donations, reflecting higher sensitivity of ID-NAT than MP-NAT screening.

**Table 1:** Blood donor NAT yield model derived West Nile Virus infection estimates, northern Texas, 2012*

Model type	No. samples tested	Person-months†	No. WNV RNA+ donations	Viremic donations/10,000 donations	Incidence/10,000 donor months	% Population infected (95% CI)	Estimated no. infections (95% CI)
MP-NAT	118,593	41,604.76	30	2.5	7.2	0.72 (0.44–1.00)	31,013 (19,133–42,893)
ID-NAT	15,134	9,725.46	24	15.9	24.7		

*NAT, Nucleic acid amplification technology †Person-months =  number of donations tested multiplied by WNV RNA detection period/30.5.

The time at risk for donors differed; detection period is estimated as 19.6 days for ID-NAT and 10.7 days for MP-NAT (Technical Appendix). The incidence rates also differed, estimated as 7.2 WNV infections (95% CI 3.5–10.9) per 10,000 donor-months (MP-NAT screening periods) and 24.7 WNV infections (95% CI 13.3–36.0) per 10,000 donor-months (ID-NAT screening periods). During the 239-day WNV season, the ratio of blood donations screened by each method was assumed to be equal to the ratio of days screened by each method (because donations per day are roughly constant throughout the season). Incidence was presumed to be 0 outside the WNV season. Applying the 4-county area’s 2011 population estimates and the number of days screened by each method to the NAT yield-derived incidence rates resulted in an estimated 31,013 WNV infections (95% CI 19,133–42,893) or 0.72% (95% CI 0.44%–1.00%) infection proportion during the 2012 epidemic season.

Of 356 probable and confirmed WNND case-patients, 7 were <15 years of age. Therefore, based on 349 probable and confirmed WNND cases, we estimated 85,156 WNV infections (95% CI 68,302–103,866) or 1.98% (95% CI 1.59%–2.41%) infection proportion during the 2012 epidemic season (Table 2). Age- and sex-based point estimates are shown in Table 2; however, these infection proportions are not statistically significant (p = 0.54), as evident by 95% CIs.

**Table 2:** West Nile neuroinvasive disease case-based model derived West Nile Virus infection estimates, northern Texas, 2012

Sex and age groups†	Total population	No. WNND case-patients (A)	Inverse ratio (B)	Estimated no. infections‡ (95% CI)	% Population infected§ (95% CI)
M	2,096,657	203	220	44,660 (33,841–56,977)	2.13 (1.61–2.72)
16–24 y	361,910	11	719	7,909 (2,357–16,723)	2.19 (0.65–4.62)
25–44 y	858,076	41	356	14,596 (7,260–24,468)	1.70 (0.85–2.85)
45–64 y	667,136	75	248	18,600 (11,210–27,851)	2.79 (1.68–4.17)
≥65 y	209,535	75	50	3,750 (1,953–6,129)	1.79 (0.93–2.92)
F	2,211,115	146	291	42,486 (28,943–58,621)	1.92 (1.31–2.65)
16–24 y	350,330	5	1,231	6,155 (626–17,397)	1.76 (0.18–4.97)
25–44 y	879,401	36	330	11,880 (5,306–21,068)	1.35 (0.60–2.40)
45–64 y	699,480	52	387	20,124 (9,949–33,847)	2.88 (1.42–4.84)
≥65 y	281,904	54	61	3,294 (1,017–6,873)	1.17 (0.36–2.44)
Total	4,307,772	349	244	85,156 (68,302–103,866)	1.98 (1.59–2.41)

*WNND, West Nile neuroinvasive disease. †Age and sex information presented for confirmed and probable case-patients >16 y of age (n = 349). ‡A × B. §No. estimated infections/population.

## Conclusions

Our findings reflect low incidence of WNV in this area; <2% of the population was infected during a large WNV epidemic, with potential incidence differences by age and sex. Low incidence was found regardless of method (NAT yield vs. WNND-based). The donor NAT yield model resulted in lower numbers of projected WNV infections in northern Texas during the 2012 arboviral season compared with the WNND-based model. These estimation differences may be caused by issues affecting internal validity in the model, resulting in overestimation or underestimation of WNV infections.

Because the donor NAT yield model used blood donors who tested WNV RNA-positive and the WNND-based model used ratios derived from blood donors, we emphasize that persons who donate blood may not reflect the total population sampling frame. Blood donors differ from the general population in age, sex, and racial and ethnic descriptions to (*10*). WNV infection rates and WNND rates also differ by age, sex, and possibly race and ethnicity (*6*). In addition, 25% of WNV-infected persons may have signs and symptoms that result in self-exclusion or deferral from blood donation (*11*). Also, the RNA detection periods on which the NAT yield model relies continues to be refined.

For the WNND-based model, although WNND cases may be more reflective of the total population sampling frame because of reporting requirements, issues with case determination and completeness of WNND reporting exist, likely resulting in underreporting. In addition, ratios used in the model were determined from North Dakota (2002–2008). This population may differ regarding exposure, disease, and reporting from that of the study population. Although interval estimation did not support differences by age and sex, possibly because of small counts, potential differences in point estimates are consistent with other observations (2,*6**,**11*)*.*

Seroprevalence studies conducted in the United States have described varying WNV infection proportions in the population after an epidemic, ranging from 2.6% to 19.7% in different geographic areas (*12*). This estimation of WNV infections in the southern United States contributes to defining the incidence of WNV infection. Despite limitations in the models, data on viremic blood donors and persons with WNND should continue to be used to determine the external validity of the models in conjunction with seroprevalence studies during outbreaks. Valid estimations of WNV infections may give insight into the overall effects of infection and could guide public health interventions in the future.

Technical Appendix Calculations and derived estimates for MP-NAT and ID-NAT detection period durations.
