# Supplementary material for: West Nile Virus Infection Incidence Based on Donated Blood Samples and Neuroinvasive Disease Reports, Northern Texas, USA, 2012
Source: Emerg Infect Dis. 2015 Apr;21(4):681–3. doi: 10.3201/eid2104.141178 (PMC4378495; doi:10.3201/eid2104.141178)
Supplement: Technical Appendix — Calculations and derived estimates for MP-NAT and ID-NAT detection period durations. [file 14-1178-Techapp-s1.pdf]

Article DOI: http://dx.doi.org/10.3201/eid2104.141178

West Nile Virus Infection Incidence Based on Donated Blood Samples and Neuroinvasive Disease Reports, Northern Texas, USA, 2012

Technical Appendix

The methods in Busch et al (*1*) have been adapted to the data from Kleinman et al (*2*). Busch et al (*1*) estimated the MP-NAT DP, but not the ID-NAT DP. The data from Kleinman et al (*2*) allows estimation of both the MP-NAT DP and the ID-NAT DP. The ID-NAT DP can be divided into three segments; the pre-MP-NAT period (denoted x_1), the MP-NAT period (denoted $\times_2$), and the post-MP-NAT period (denoted $\times_3$). Thus,

$$\text{ID-NAT DP}=\chi_1 + \chi_2+\chi_3$$

and

$$\text{MP-NAT DP} = \chi^2$$

Busch et al (*1*) estimated the MP-NAT DP to be 6.9 days (s.e. 2.0 days), but not the ID-NAT DP. Instead, results from Busch et al (*3*) and Kleinman et al (*2*) are used to estimate concurrently the MP-NAT DP and ID-NAT DP. Busch et al (*3*) estimated the time from RNA MP positivity to RNA ID negativity to be 13.2 days (s.e. 1.0 days). Assuming RNA MP positivity of enrolled donors in Busch et al could occur anytime in the MP-NAT DP, then 13.2 days represents the mid-point of the MP-NAT DP until the end of the ID-NAT DP;

$$1/2\ \chi_2 + \chi_3 = 13.2$$

Kleinman et al (*2*) found among 75 confirmed WNV positive donors detected by ID-NAT testing, 4 donated in the pre-MP-NAT period, 41 donated in the MP-NAT period, and 30 donated

in the post-MP-NAT period. Assuming these donors are equally likely to attempt donation anytime during the ID-NAT DP, then the three periods are in the ratio;

$$\chi_1 : \chi_2 : \chi_3 = 4:41:30$$

or equivalently:

$$\chi_1/\chi_2=4/41$$

$$\chi_3/\chi_2=30/41$$

Solving algebraically for the three segments:

$$\chi_1=1.0$$

$$\chi_1=1.0$$

$$\chi_2=10.7$$

$$\chi_3=7.9$$

Hence the ID-NAT DP is estimated to be 19.6 days, and the MP-NAT DP is estimated to be 10.7 days.

The standard errors for these two DP estimates can be approximated using Taylor series expansion (*4*) based on the standard error of the 13.2 day estimate, and the trinomial distribution for the division of the 75 ID-NAT donors into three segments. The standard error estimates are 2.3 days for ID-NAT DP and 2.0 days for MP-NAT DP.

References

1. Busch MP, Wright DJ, Custer B, Tobler LH, Stramer SL, Kleinman SH, Prince HE, Bianco C, Foster G, Petersen LR, Nemo G, and Glynn SA. West Nile virus infections projected from blood donor screening data, United States, 2003. Emerg. Infect. Dis.2006; 12(3): 395–402. PubMed

2. Kleinman SH, Williams JD, Robertson G, Caglioti S, Williams RC, Spizman R, et al. West Nile virus testing experience in 2007: evaluation of different criteria for triggering individual-donation nucleic acid testing. Transfusion. 2009;49:1160–70. PubMed http://dx.doi.org/10.1111/j.1537-2995.2009.02127.x

3. Busch MP, Kleinman SH, Tobler LH, Kamel HT, Norris PJ, Walsh I, et al. Virus and Antibody Dynamics in Acute West Nile Virus Infection. J Infect Dis. 2008;198:984–93 http://dx.doi.org/10.1086/591467. PubMed

4. Miller RG. Survival Analysis. New York: John Wiley; 1981.
